# *Schinus terebinthifolia* leaf lectin (SteLL) has anti-infective action and modulates the response of *Staphylococcus aureus-*infected macrophages

**DOI:** 10.1038/s41598-019-54616-x

**Published:** 2019-12-03

**Authors:** Isana Maria de Souza Feitosa Lima, Adrielle Zagmignan, Deivid Martins Santos, Hermerson Sousa Maia, Lucas dos Santos Silva, Brenda da Silva Cutrim, Silvamara Leite Vieira, Clovis Macêdo Bezerra Filho, Eduardo Martins de Sousa, Thiago Henrique Napoleão, Karen Angeliki Krogfelt, Anders Løbner-Olesen, Patrícia Maria Guedes Paiva, Luís Cláudio Nascimento da Silva

**Affiliations:** 1 Programas de Pós-Graduação, Universidade Ceuma, São Luís, Maranhão Brazil; 2 Departamento de Bioquímica, Universidade Federal de Pernambuco, Recife, Pernambuco Brazil; 3 Department of Viral and Microbial Diagnostics, Statens Serum Institut, Copenhagen, Denmark; 4 Department of Science and Environment, Roskilde University, 4000 Roskilde, Denmark; 5 Department of Biology, Section for Functional Genomics, University of Copenhagen, Copenhagen, Denmark

**Keywords:** Lectins, Applied microbiology

## Abstract

*Staphylococcus aureus* is recognized as an important pathogen causing a wide spectrum of diseases. Here we examined the antimicrobial effects of the lectin isolated from leaves of *Schinus terebinthifolia* Raddi (SteLL) against *S. aureus* using *in vitro* assays and an infection model based on *Galleria mellonella* larvae. The actions of SteLL on mice macrophages and *S. aureus-*infected macrophages were also evaluated. SteLL at 16 µg/mL (8 × MIC) increased cell mass and DNA content of *S. aureus* in relation to untreated bacteria, suggesting that SteLL impairs cell division. Unlike ciprofloxacin, SteLL did not induce the expression of *recA*, crucial for DNA repair through SOS response. The antimicrobial action of SteLL was partially inhibited by 50 mM *N*-acetylglucosamine. SteLL reduced staphyloxathin production and increased ciprofloxacin activity towards *S. aureus*. This lectin also improved the survival of *G. mellonella* larvae infected with *S. aureus*. Furthermore, SteLL induced the release of cytokines (IL-6, IL-10, IL-17A, and TNF-α), nitric oxide and superoxide anion by macrophagens. The lectin improved the bactericidal action of macrophages towards *S. aureus*; while the expression of IL-17A and IFN-γ was downregulated in infected macrophages. These evidences suggest SteLL as important lead molecule in the development of anti-infective agents against *S. aureus*.

## Introduction

*Staphylococcus aureus* is recognized as an important pathogen causing a wide spectrum of diseases including cutaneous and blood stream infections^1,2^. This versatility is ensured by the high ability of this microorganism to acquire drug resistance and to produce virulence factors that are regulated by complex genetic networks^2–5^. The multiple virulence factors identified in *S. aureus* play different roles during the infection such as adhesion, host lesions and evasion of the immune system, even from professional phagocytes such as macrophages^6–8^.

It has been reported that the dissemination of *S. aureus* is associated to the capacity of this bacterium to survive and replicate inside the phagocytes (in both phagosome and/or cytoplasm), and modulate important cellular mechanisms such as autophagy, apoptosis and pyronecrosis^9–11^. *S. aureus* is also able to release several effector molecules to suppress or enhance cytokine production (including IL-1β, IL-17 and TNF) as well as to damage immune cells and host tissues^6,12–14^. Thus, compounds able to improve and/or regulate the cellular immune response have been pointed out as promising lead drugs for treatment of microbial infections and also to improve the general understanding related to host-pathogen interactions^15–19^.

Plants are important sources of molecules to be used for drug development due their high chemical variability and diverse action mechanisms^20,21^. Among plant derived compounds, lectins have displayed a wide range of biotechnological applications, including antimicrobial and immunomodulatory actions^16,19,22–27^. Previously, the isolation of SteLL, an *N*-acetylglucosamine-binding (NAG) lectin, from leaves of *Schinus terebinthifolia* Raddi (Anacardiaceae) was reported.

SteLL is a 14 kDa glycoprotein and the antimicrobial activity of this protein was reported towards both Gram-positive and Gram-negative bacteria and *Candida albicans*^25^. SteLL also affected the survival and nutrition of the beetle *Sitophilus zeamais* adults^28^. Recently, the antitumoral activity of SteLL was shown in sarcoma 180-bearing mice. The authors reported that the treatment with SteLL did not induce hematological changes nor genotoxic effects in mice, advocating for the safety of *in vivo* use of this lectin^29^.

This present work provides insights into the *in vitro* effects of SteLL on *S. aureus* and evaluates the phenotypic response induced by this lectin in macrophages uninfected and/or infected by *S. aureus*. In addition, the *in vivo* activity of this lectin against *S. aureus* is reported using *Galleria mellonella* larvae (Lepidoptera: Pyralidae) as infection model.

## Results

### SteLL induced changes in the cell size/DNA content ratio of *S. aureus*

**Figure 1:**
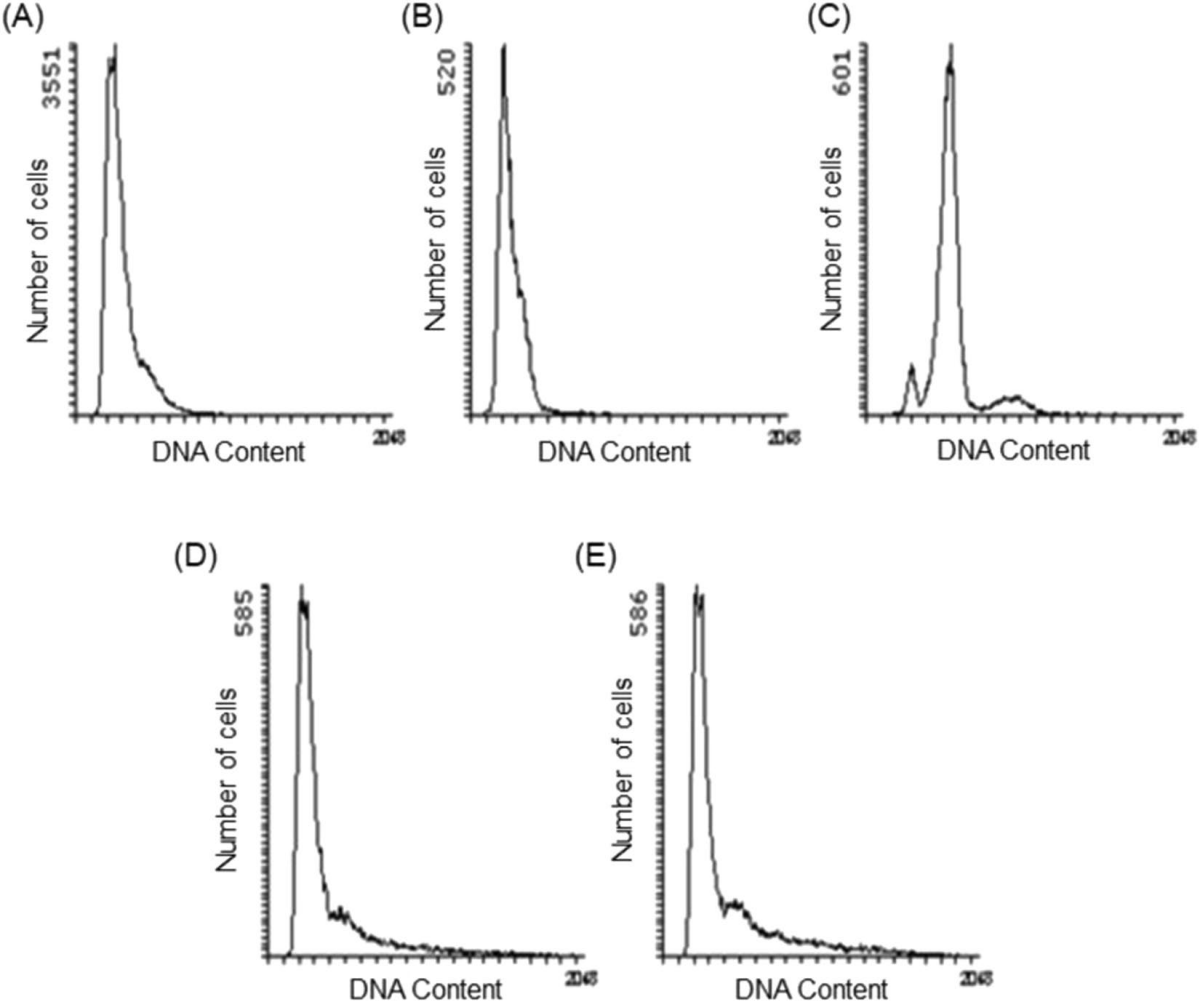
Effects of SteLL and selected antimicrobials on DNA content of *S. aureus* 8325-4. (**A**) Exponentially growing cells; (**B**) Cells treated with 0.78 µg/mL ciprofloxacin for 3 h; (**C**) Cells treated with 12.5 µg/mL chloramphenicol for 3 h; (**D**) Cells treated with 2 × MIC SteLL (4 µg/mL) for 3 h; (**E**) Cells treated with 8 × MIC (16 µg/mL) SteLL for 3 h.

As previously reported^25^, SteLL was able to inhibit the growth of all *S. aureus* strains tested (*S. aureus* 8325-4, *S. aureus* ATCC 6538, *S. aureus* ATCC 29312) with a MIC (minimum inhibitory concentration) of 2 µg/mL. A flow cytometry-based assay was performed to assess the effects of SteLL on cell size (seen by mean light scattering detected in LS1) and DNA content (seen by the fluorescence intensity in FL2 channel) of *S. aureus*. Ciprofloxacin (inhibitor of DNA replication) and chloramphenicol (inhibitor of protein synthesis) were used as controls in this assay (Table 1 and Fig. 1).

**Table 1:** Effects of SteLL on cell size and DNA content of *S. aureus* 8325-4 during exponential growth.

	Untreated cells	CAM (1 × MIC)	CIP (1 × MIC)	SteLL (2 × MIC)	SteLL (8 × MIC)
Cell mass	426.1 ± 36.1^a^	470.0 ± 26.6^a^	638.0 ± 44.7^b^	492.3 ± 62.2^a^	560.6 ± 65.2^b^
DNA content	303.8 ± 41.5^a^	562.2 ± 31.0^b^	264.4 ± 36.4^a^	383.1 ± 73.1^a^	422.5 ± 74.4^b^
DNA/mass ratio	0.71	1.20	0.41	0.78	0.75

Cell mass and DNA content was measured by flow cytometry using forward scattering (at LS1 detector) and fluorescence intensity (at FL2 channel) and are expressed by arbitrary units (a.u.). Legend: CAM: Chloramphenicol; CIP: Ciprofloxacin; SteLL: *Schinus terebinthifolia* leaf lectin. In each row the values with significant differences (p < 0.05) are indicated by different letters.

The exposure of *S. aureus* to ciprofloxacin at MIC resulted in significantly increase in cell mass, while the DNA content decreased when compared with untreated cells. This resulted in a decrease in the cellular DNA concentration (DNA/mass ratio = 0.41) compared to untreated bacteria (DNA/mass ratio = 0.71). Bacteria treated with chloramphenicol did not alter cell size as cell division requires protein synthesis and hence was blocked. In these bacteria, the cellular DNA content increased (DNA/mass ratio = 1.20), which is consistent with a DNA replication arrest specifically at the level of initiation. Consequently, runout chromosome synthesis was observed and the bacterial cells end up with integral numbers of fully replicated chromosomes (Fig. 1C).

**Figure 2:**
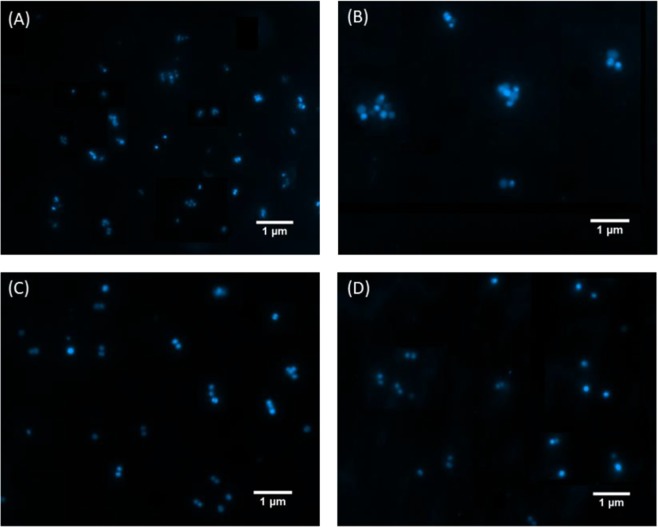
Effects of SteLL on cell size of *S. aureus* 8325-4. (**A**) Exponentially growing cells; (**B**) Cells treated with 0.78 µg/mL ciprofloxacin for 3 h; (**C**) Cells treated with 2 × MIC SteLL (4 µg/mL) for 3 h; (**D**) Cells treated with 8 × MIC (16 µg/mL) SteLL for 3 h.

Cells treated with SteLL did not follow any of these profiles (Fig. 1D,E). A significant increase (31%) on cell size was observed in bacteria treated with SteLL at 8 × MIC (16 µg/mL), this was accompanied by a similar increase in DNA content (39%), and consequently the DNA concentration remained almost unchanged in these cells (DNA/mass ratio = 0.75) (Table 1). The effects of SteLL on *S. aureus* cell size were also confirmed by fluorescence microscopy (Fig. 2), where cells treated with the lectin (Fig. 2C,D) appeared with increased size when compared with control cells (Fig. 2A).

**Figure 3:**
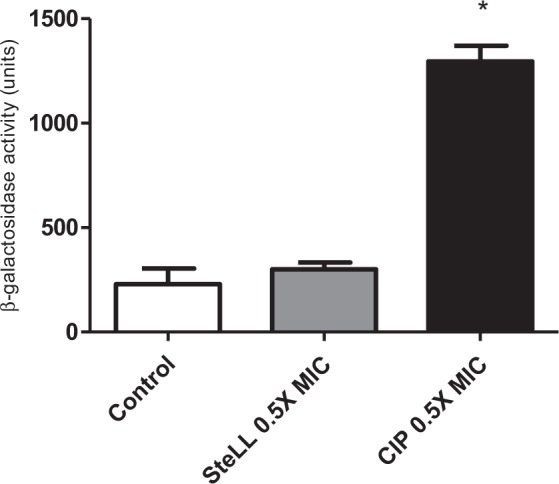
Effects of SteLL on *recA* expression of *S. aureus*. The expression of *recA* were performed using a derivative *S. aureus* 8325-4 strain carrying a *recA::lacZ* fusion. β-galactosidase activity was measured using ONPG. (*) Indicates significant differences in relation to control cells (p < 0.05).

We also evaluated whether sub-inhibitory concentration of SteLL (0.5 × MIC) had any effect on the SOS response, a pathway associated with acquisition of drug resistance and virulence phenotypes. After 3 h of treatment, *recA* transcription determined from a *recA-lacZ* transcriptional fusion was not altered by SteLL (Fig. 3). On the other hand, ciprofloxacin (a DNA damaging agent) induced more than 5-fold increase in *recA* transcription. These results revealed that SteLL, unlike ciprofloxacin, did not affect DNA integrity and thus did not induce any SOS-related mutagenic pathways.

### SteLL increased ciprofloxacin activity against *S. aureus*

Checkerboard assays were performed to evaluate the interaction of SteLL with two selected antibiotics: ciprofloxacin and ampicillin. The MIC values for these two drugs towards *S. aureus* 8325-4 were 0.78 µg/mL and 25 µg/mL, respectively. A synergistic activity was observed when combining SteLL with ciprofloxacin (ΣFIC: 0.47); while an additive effect was found between SteLL and ampicillin (ΣFIC: 0.53).

**Figure 4:**
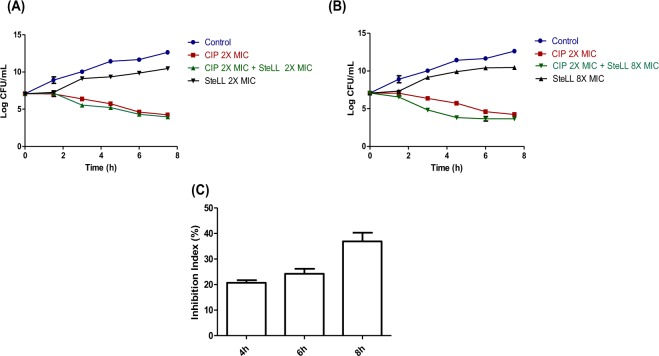
Antimicrobial action of SteLL against *S. aureus*. (**A**) Time-kill curves for 2 × MIC SteLL (4 µg/mL) alone or in combination with 2 × MIC ciprofloxacin. (**B**) Time-kill curves for 8 × MIC SteLL (16 µg/mL) alone or in combination with 2 × MIC ciprofloxacin. (**C**) Effects of N-acetylglucosamine (NAG) on antimicrobial action of 8 × MIC SteLL (16 µg/mL).

We subsequently performed time-kill assays on *S. aureus* strain 8325-4 using SteLL (2 × MIC and 8 × MIC), ciprofloxacin (2 × MIC), and combinations of these two agents (2 × MIC SteLL + 2 × MIC ciprofloxacin and 8 × MIC SteLL + 2 × MIC ciprofloxacin). Both SteLL concentrations delayed the onset of growth. After 7.5 h, the reductions of bacterial counts by both SteLL concentrations were approximately 2 log CFU/mL (relative to untreated cells). In contrast, ciprofloxacin (2 × MIC) strongly reduced the bacterial growth in a time-dependent manner (Fig. 4A,B).

When SteLL was combined at 2 × MIC, it did not induce any effect on ciprofloxacin (2 × MIC) bactericidal action (ΔLC were less than 1 log CFU/mL when compared with ciprofloxacin-treated cells). On the other hand, the combination of ciprofloxacin at 2 × MIC and SteLL at 8 × MIC was more effective than ciprofloxacin alone. For this combination, significant reductions were observed at all evaluated times, with the maximal effect observed after 4.5 h of incubation. At this time a ΔLC of 1.38 log CFU/mL relative to cells treated with ciprofloxacin alone. Thus, at this concentration SteLL exhibited an additive bactericidal effect resulting in a faster reduction in viable bacteria during the first hours of incubation (Fig. 4B).

In order to evaluate the involvement of carbohydrate-binding domain, we compared the effects of SteLL (8 × MIC) on *S. aureus* growth in the presence of 50 mM NAG. The antimicrobial action of SteLL was partially inhibited by NAG with inhibition index (IN%) of 20.67 ± 1.07, 24.19 ± 1.97 and 36.91 ± 3.35 after incubation of 4 h, 6 h and 8 h (Fig. 4C).

### SteLL inhibits staphyloxanthin production

**Figure 5:**
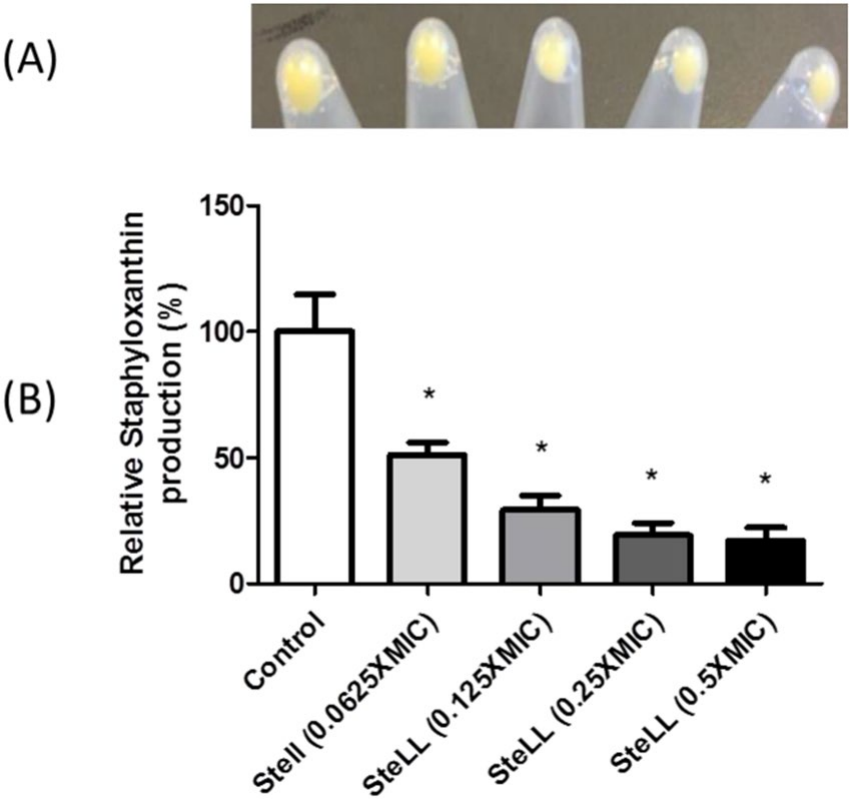
Effects of SteLL on staphyloxanthin production by *S. aureus* ATCC 29312. (**A**) Qualitative assay; (**B**) Quantitative assay. (*) Indicates significant differences in relation to control cells (p < 0.05).

Next, we evaluated whether sub-inhibitory SteLL concentrations (0.065 × MIC, 0.125 × MIC, 0.25 × MIC and 0.5 × MIC) could inhibit the production of staphyloxanthin, the golden pigment of *S. aureus* ATCC 29312. SteLL induced a dose-dependent reduction in staphyloxanthin production relative to untreated bacteria (Fig. 5A). The reductions ranged from 48.78% to 82.88% (Fig. 5B). The treatment with the highest tested concentration of SteLL (0.5 × MIC) reduced the staphyloxanthin content to around 18% (in comparation to untreated bacteria), resulting in almost colorless *S. aureus* cells.

### SteLL increased the release of nitric oxide and superoxide by mice macrophages

**Figure 6:**
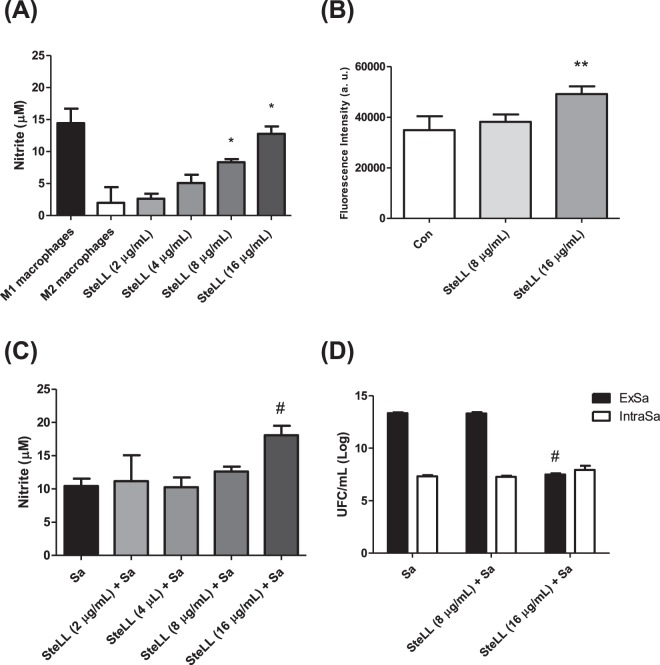
Effects of SteLL on different responses of mice peritoneal macrophages. (**A**) Nitric oxide release by mice macrophages induced by SteLL. (**B**) Production of mitochondrial superoxide anion by mice peritoneal macrophages induced by SteLL. (**C**) Nitric oxide release by *S. aureus-*infected macrophages induced by SteLL. (**D**) Effects of SteLL on bactericidal activity of mice peritoneal macrophages towards extracellular and (ExSa) intracellular (IntraSa) *S. aureus*. M1 macrophages: macrophages treated with LPS + INF-γ. M2 macrophages: macrophages treated with IL-4 + IL-13. Con: untreated cells; Sa: *S. aureus*. (*) Indicates significant differences in relation to M2 macrophages (p < 0.05). (**) Indicates significant differences in relation to untreated macrophages (p < 0.05). (#) Indicates significant differences in relation to *S. aureus*-infected cells without SteLL treatment (p < 0.05).

The macrophage viability was not affected by any tested SteLL concentrations (2–16 µg/mL) (data not shown). On the other hand, the treatment of macrophages with different concentrations of this lectin resulted in a significant increase in NO production in relation to untreated cells (Fig. 6A). Maximum NO production was observed in the presence of SteLL at 8 µg/mL and 16 µg/mL. At these concentrations, the levels of NO were similar (p > 0.05) to those produced by M1 macrophages.

The induction of mitochondrial superoxide by SteLL was evaluated using the MitoSOX fluorescent probe. The macrophages were incubated for 30 min with SteLL at 8 µg/mL and 16 µg/mL. As shown in Fig. 6B, only the treatment with SteLL at 16 µg/mL significantly enhanced superoxide production by the macrophages (increase of 40%).

### SteLL increased the bactericide activity of mice macrophages

Next, we evaluated the effects of SteLL on *S. aureus*-infected macrophages. SteLL at 16 µg/mL significantly increased the NO levels released by *S. aureus*-infected macrophages (p > 0.05) (Fig. 6C). The highest tested concentration of SteLL (16 µg/mL) also induced a significant decrease in bacterial load in the supernatant (7.49 ± 0.33 log CFU/mL) in relation to untreated *S. aureus*-infected macrophages (13.35 ± 0.24 log CFU/mL) (Fig. 6D). However, the intracellular amount of *S. aureus* was not altered by SteLL treatment (Fig. 6D). The same response was observed in macrophages infected with *S. aureus* ATCC 6538 and treated with SteLL (Supplementary Fig. 1). These results indicated that the reduction of bacteria in supernatant of SteLL-treated macrophages is related with the increased release of reactive species.

### SteLL modulated cytokine release by uninfected macrophages or *Staphylococcus aureus*-uninfected macrophages

**Figure 7:**
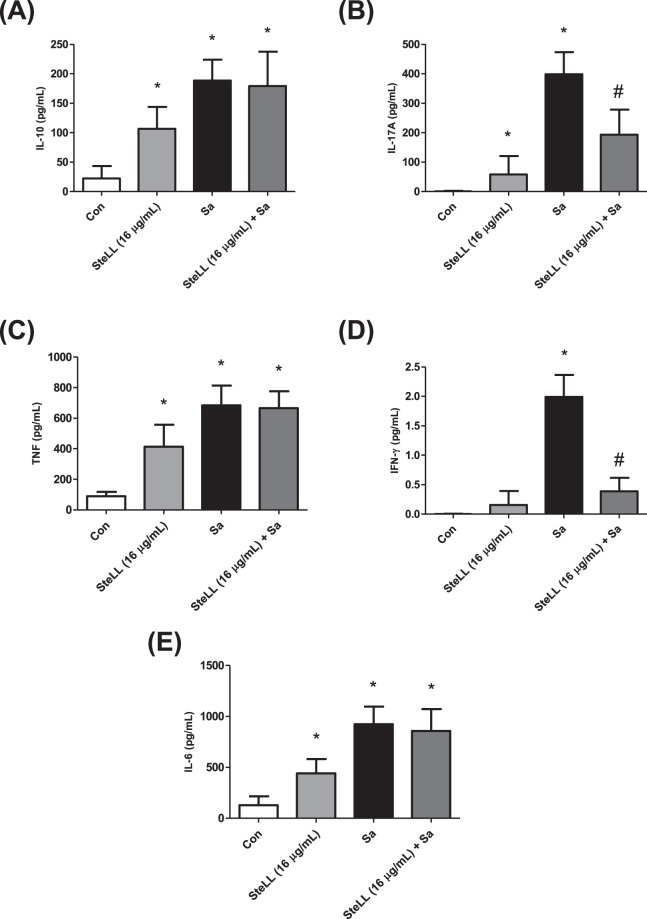
Effects of SteLL on cytokine release of mice peritoneal macrophages infected or not with *S. aureus*. (**A**) IL-10; (**B**) IL-17A; (**C**) TNF; (**D**) INF-γ; (**E**) IL-6. Con: untreated cells; Sa: *S. aureus*. (*) Indicates significant differences in relation to control cells (p < 0.05). (#) Indicates significant differences in relation to *S. aureus* infected cells (p < 0.05).

We selected the most active concentration of SteLL (16 µg/mL) to evaluate its influence on cytokine release pattern of macrophages uninfected and infected with *S. aureus*. For uninfected macrophages, the treatment with this dose resulted in a significant enhancement of IL-6, IL-10, IL-17A, and TNF-α compared to untreated cells (p > 0.05) (Fig. 7A,B,C,E). The levels of IFN-γ were also higher in the supernant of SteLL-treated cells, although no statitical differences were found when compared with the values obtained for control cells (Fig. 7D). The lectin did not influence the levels of IL-4 and IL-12 (data not shown).

The macrophages infected with *S. aureus* expressed high levels of IL-6, IL-10, IL-17A, and TNF-α (Fig. 7). The roles of theses cytokines in pathogenesis of *S. aureus* have been described previously^30–32^. The level of IFN-γ was also increased by *S. aureus* infection (about 2-fold). SteLL was able to downregulate the expression of IL-17A (Fig. 7B) and IFN-γ (Fig. 7D) by infected macrophages, while the levels of the other tested cytokines were also reduced albeit not in a significant manner. It is important to highlight that SteLL did not totally inhibit the release of IL-17A and IFN-γ; in fact, their levels were reduced to approximately what was found in the supernatant of control cells.

### SteLL protected *Galleria mellonella* larvae against *Staphylococcus aureus* infection

To finally show the treatment efficacy of SteLL, we employed an infection assay using *G. mellonella* larvae. This model has been widely used to study microbial pathogenesis^33^ and to assess the *in vivo* activity of antimicrobial agents^34,35^. Uninfected larvae inoculated with PBS or SteLL exhibited similar survival curves (p > 0.05). On the other hand, infection with *S. aureus* 8325-4 reduced the larval viability by 30%, 80% and 100%, on day 1, 2 and 3, respectively. The median survival of this group was 2 days.

**Figure 8:**
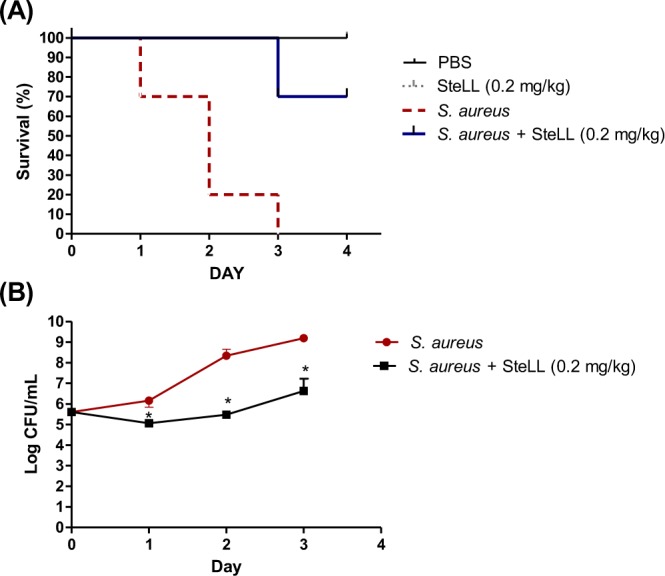
Effects of SteLL on survival (**A**) and bacteria load in hemolymph (**B**) of *G. mellonella* larvae infected with *S. aureus*. In all experiments the larvae were infected with a *S. aureus* 8325-4 suspension (10 μL of 1.0 × 10^5^ CFU/mL) and treated with SteLL at 0.2 mg/kg. (*) Indicates significant differences in relation to control cells (p < 0.05).

The single-dose treatment using 0.2 mg/kg SteLL (corresponding to the administration of 10 µL of a SteLL solution at 2 × MIC) increased the survival of *S. aureus*-infected insects. For this group, the median survival was not possible to be defined, and only a 30% reduction in survival was recorded after 3 days (Fig. 8A). The survival curves of SteLL-treated larvae and *S. aureus-*infected group were significantly different (p < 0.05).

Next, we evaluated the number of *S. aureus* colonies in the hemolymph of larvae. The untreated larvae infected with *S. aureus* exhibited increased levels of bacteria in hemolymph during the experiment (Fig. 8B). The treatment with SteLL was able to significantly inhibit the bacteria growth (p < 0.05). During the first two days the bacterial load in SteLL-treated animals remained the same as at time of inoculation and only a small increase was observed in the third day (about 1 log CFU/mL). This effect is in accordance to the effect of SteLL in the time kill assay (Fig. 3). In this sense, SteLL treatment reduced the *S*. *aureus* proliferation in larvae hemolymph resulting in increased animal survival.

## Discussion

In this study, we evaluated the *in vitro* effects of SteLL on *S. aureus* and the responses induced by this protein in two models of infections (using macrophages and *G. mellonella*). SteLL is purified from leaves of *S. terebinthifolia*, a medicinal plant widely used in Northeastern Brazilian (where it is popularly known as “Aroeira da praia”) to treat skin wounds and inflammation^36^. Besides SteLL, other products derived from *S. terebinthifolia* have exhibited antimicrobial activity such as essential oils and some purified compounds^37–39^.

We employed a flow cytometry-based method to analyze whether SteLL could induce any effect on cell size and DNA content^40^. Proper cell cycle control is essential to ensure the generation of two identical daughter cells as result of cell division^41–43^. Perturbations in cell cycle regulation are therefore deleterious for bacterial proliferation; and thus the proteins involved in this pathway constitute potential targets for drug action^44^. We observed that SteLL induced significant increases in both the cell size and DNA content after 3 hours of incubation. These effects were confirmed by fluorescence microscopy and may indicate impairment of cell division (since both DNA and cell mass are increased), a kind of response that has been described for other antimicrobial agents such as targocil, a cell wall stressor^45^.

The ability to bind *N*-acetylglucosamine residues has been associated with the bacteriostatic properties of chitin-binding lectins (such as SteLL)^25,26,46^, since high amount of peptidoglycan in *S. aureus* cell wall provides multiples targets for interactions. We demonstrated that 50 mM NAG partially inhibited the antimicrobial action of SteLL. Thus, it is possible to hypothesize that SteLL binds *N*-acetylglucosamine residues present in cell wall and disturbs the process of cell division.

In addition, SteLL treatment did not induce the expression of *recA* which is instrumental in triggering the SOS response. RecA detects ssDNA generated by DNA degradation or inhibition of DNA replication, stimulates LexA autocleavage which in turn leads to derepression of a number of LexA regulated genes. These genes encode enzymes involved in DNA repair and mutagenesis (reviewed by Simmons *et al*.^47^). Taken together, these findings indicated that SteLL may inhibit bacterial growth by impairing division without affecting DNA structure.

Since SteLL inhibits the growth of *S. aureus*, we evaluated whether this lectin could affect the activity of antibiotics in clinical use. The combinatory effects of SteLL and the antibiotics ciprofloxacin or ampicillin were assessed using checkboard and time-kill experiments. Initially, we found that this lectin improved the action of ampicillin (β-lactam) and ciprofloxacin (quinolone) through additive and synergistic effects, respectively. SteLL at 16 µg/mL (8 × MIC) could also increase the bactericidal properties of ciprofloxacin, however, this action was only observed during the first hours. The synergistic interactions with antibiotics have been reported only for few plant lectins, including the lectins extracted from *Alpinia purpurata* (ApuL)^24^ and *Vatairea macrocarpa* (VML)^48^.

Another effect of SteLL on *S. aureus* physiology is the inhibition of staphyloxanthin production, a carotenoid pigment encoded by the *crtOPQMN* operon. Staphyloxanthin has been associated with the protection against oxidant attack promoted by immune cells^49^, which brought a new light in the use of this pigment as target for drug development^34,50^. Some plant derived compounds have inhibitory effects on staphyloxanthin^34,51,52^, however, this is the first report of a similar action for a plant lectin.

Given the well known ability of plant lectins to alter the phenotic responses of immune cells, we examined the effects of SteLL on macrophages and *S. aureus*-infected macrophages. The results showed that SteLL induced the the release of cytokines (IL-6, IL-10, IL-17A, and TNF-α) and reactive species (nitric oxide, superoxide anion) by uninfected macrophagens. The ability of plant lectins to alter the macrophage responses have been demonstrated by other authors^18,19,53^. The effects of SteLL on nitric oxide and superoxide anion production may be related to the improvement of macrophages bactericidal action, since these reactive species play essential roles in the defense against infectious diseases^54,55^.

Importantly, SteLL modulated the expression of two proinflammatory cytokines (IL-17A and IFN-γ) in infected macrophages. In the context of *S. aureus* infectious, IL-17A is essential for antimicrobial peptides production and bacterial clearance^56^, while IFN-γ increase the macrophages response against *S. aureus*^57^. Intriguingly, the overproduction of both cytokines can also exacerbate the severity of some infections^14,58^. For example, *S. aureus* phenol-soluble modulins that induce high levels of IL-17 leading to skin inflammatory response^13^.

The excess of IFN-γ is also related to the harmful inflammation state associated with the damage of essential organs (such as liver and kidney)^59^. Recent evidences suggested that IFN-γ favored the outgrowth of *S. aureus*^58^. Thus, inhibition of the expression of both IFN-γ and IL-17A has been reported as a beneficial effect in several models of *S. aureus-*induced infection^59,60^.

Although the molecular mechanism underlying the suppression of IL-17A and IFN-γ induced by lectin treatment in macrophages infected with *S. aureus* remains to be elucidated, it is possible that decreased levels of these cytokines help to attenuate the deleterious effects of persistent inflammatory responses at the site of infection. Furthermore, because the non-infected cells treated with SteLL presented different cytokine profiles than those found in the *S. aureus*-infected cells treated with SteLL, it is likely that bacterial components also contribute to modulation of the macrophage response.

These paradoxical effects on the production of inflammatory mediators when comparing uninfected and infected hosts have been observed for other plant lectins^16,18,19^. For instance, the lectin isolated from *Cratylia mollis* (Cramoll) has been described as pro-inflammatory agent in *in vitro* and *in vivo* models^18,61–63^; however the treatment with this protein was shown to reduce the release of cytokines (such as TNF-α, IL-6) in experimental models of infection induced by *S. aureus* (using peritoneal cells)^18^ and *Cryptococcus gatti* (using mice)^64^.

Similarly, the lectin from *Canavalia brasiliensis* (ConBr) induced different responses in *Salmonella enteritidis*-infected and uninfected macrophages. The exposition of uninfected macrophages to ConBr resulted in high levels of mRNA transcripts for IL-6 (in relation to untreated and uninfected macrophages). However, *S. enteritidis-*infected macrophages treated with this lectin exhibited lower levels of IL-6 gene transcription when compared to untreated *S. enteritidis*-infected cells. ConBr treatment also suppressed the transcription of IL-10 gene in macrophages infected with *S. enteritidis*^19^.

## Material and Methods

### *S. aureus* strains

*S. aureus* 8325-4, *S. aureus* ATCC 6538 and *S. aureus* ATCC 29312 were used for antimicrobial evaluation. The *S. aureus* 8325-4 derivative strain carrying a *recA::lacZ* transcriptional fusion in its chromosome^65^ was kindly shared by Prof. Dr. Hanne Ingmer. The *S. aureus* ATCC 29312 was used for staphyloxanthin quantification, since *S. aureus* 8325-4 is a weak producer due a natural deletion in *rsbU*^66^.

### Lectin purification

SteLL was purified from dried leaves of *S. terebinthifolia* (collected in Recife, Brazil) using the methodology reported by Gomes *et al*.^25^. The leaves were obtained from specimens grown in the campus of the ‘Universidade Federal de Pernambuco’ at Recife, Brazil (8°02′55.9″S 34°56′48.4″W). The plant collection was authorized by ‘Instituto Chico Mendes de Conservação da Biodiversidade’ from Brazilian Ministry of Environment (license number: 36301).

For SteLL purification, the powder from dried leaves (20 g) was suspended in a saline solution (0.15 M NaCl) and submitted to agitation at 4 °C. After 16 h, the filtered extract was centrifuged (3000 × *g* for 15 min) and then submitted to chitin column (Sigma-Aldrich, MO, USA). The elution was performed using acetic acid (1 M) and SteLL was obtained after dialysis (10 kDa cut-off membrane; Sigma-Aldrich) against distilled water (4 h, 4 °C) and in sequence against 0.15 M NaCl (4 h, 4 °C). The protein concentration was determined according to Lowry *et al*. using a standard curve of bovine serum albumin (31.25‒500 μg/mL)^67^.

### Antibacterial activity and combinatory effects with antibiotics

#### MIC determination

The antimicrobial activity of SteLL was confirmed by determination of the Minimum Inhibitory Concentration (MIC) against *S. aureus* strains (*S. aureus* 8325-4, *S. aureus* ATCC 6538 and *S. aureus* ATCC 25923) using broth microdilution assay^25^. Briefly, serial dilutions of SteLL were prepared in 96-wells plates containing Luria-Bertani (LB) broth to obtain concentrations ranging from 128 to 0.25 µg/mL. Following, each well received 10 μL of a microbial suspension (resulting in a bacterial load of approximately 1.0 × 10^7^ CFU/mL for each well). Bacterial growth was detected measuring the optical density at 600 nm (OD_600_).

The antimicrobial action of SteLL was also evaluated in the presence of NAG, to evaluate the participation of carbohydrate-binding domain. For this, SteLL (16 µg/mL) was pre-incubated with 50 mM NAG. After 1 h, the bacteria were added as described for MIC determination. The bacterial growth was determined after 3 h, 6 h and 9 h of incubation. The inhibition index (IN%) was calculated using the following equation:

$${\rm{Inhibition}}\,{\rm{index}}({\rm{IN}} \% ):100-[({{\rm{BAC}}}_{{\rm{SteLL}}}\times 100/{\rm{BAC}})/({{\rm{BAC}}}_{{\rm{SteLL}}+{\rm{NAG}}}\times 100/{{\rm{BAC}}}_{{\rm{NAG}}})]$$

Where, BAC is the growth of untreated bacteria; BAC_SteLL_ is the growth of bacteria in the presence of SteLL; BAC_NAG_ is the growth of untreated bacteria in presence of NAG; BAC_SteLL+NAG_ BAC_SteLL_ is the growth of bacteria in the presence of SteLL and NAG.

#### Combinatory effects

The interaction between SteLL and drugs (ciprofloxacin or ampicillin) were evaluated using checkerboard assay. Fractional inhibitory concentration index (FICI) was assessed algebraically by the sum of the single FIC values for each sample present in the well:

$${\rm{FICI}}={{\rm{FIC}}}_{{\rm{L}}}+{{\rm{FIC}}}_{{\rm{D}}}=({\rm{L}}/{{\rm{MIC}}}_{{\rm{L}}})+({\rm{D}}/{{\rm{MIC}}}_{{\rm{D}}})$$

where “L” is the concentration (µg/mL) of SteLL in a given well, and MIC_L_ represents the control MIC of SteLL alone. “D” is the concentration of the tested drug in a given well, and MIC_D_ represents the control MIC of the tested drug alone. FICI mean (ΣFIC) is derived by averaging the FICI values along the growth–no growth interface. Data interpretation: ΣFIC ≤ 0.5: synergism (syn); 0.5 < ΣFIC ≤ 1: addition (add); 1 < ΣFIC < 4: noninteraction (non); ΣFIC ≥ 4: antagonism (ant)^68^.

#### Time-kill studies

Overnight cultures of *S. aureus* 8325-4 were diluted 1:100 in LB broth and placed in a shaking water bath at 37 °C. When the cultures reached an OD_600_ of 0.1 they were distributed in fresh LB broth containing increasing concentrations of SteLL (2 × and 8 × MIC) or ciprofloxacin (2 × MIC) alone or combined. The cell growth was monitored by plating 4 µL of 10-fold-diluted suspensions from each tube in quadruplicate and following the OD_600_ at 0, 1.5 h, 3.0 h, 4.5 h, 6.0 h and 7.5 h. The plates were incubated at 37 °C for 24 h. After this period, the colonies were counted for the calculation of CFU/mL. The bactericidal combinatory effects was assessed by variation on Log CFU/mL (ΔLC) and the effects were recorded as synergistic if ΔLC ≥ 2 log CFU/mL; additive if ΔLC was between 1 and 2 log CFU/mL); indifference if ΔLC = ±1 log CFU/mL; or antagonism if ΔLC > −1 log CFU/mL^69^.

### Effects of SteLL on cell size and DNA content

The possible effects of SteLL on cell size and DNA content of *S. aureus* were evaluated using flow cytometry and fluorescence microcopy. For flow cytometry, exponentially growing cells of *S. aureus* 8325-4 were treated with SteLL (at 4 µg/mL and 16 µg/mL, corresponding to 2 × MIC and 8 × MIC, respectively), ciprofloxacin (0.78 µg/mL; 1 × MIC), or chloramphenicol (12.5 µg/mL; 1 × MIC) for 3 h. Bacteria were centrifuged (9000 × *g* for 8 min at 4 °C) and fixed by resuspending in 100 µL Tris (10 mM; pH 7.5) and adding 1 mL of ethanol (77%). For staining, each sample (100 µL) was centrifuged as above and the cell pellet was then resuspended in 170 µL of a solution of ethidium bromide (20 µg/mL) and mithramycin (90 µg/mL). For each sample, a minimum of 15,000 cells were analyzed using an Apogee A10 instrument. Cell mass and DNA content were determined by the measurement of forward scattering (at LS1 detector) and fluorescence intensity (at FL2 channel), respectively^40^.

For fluorescence microscopy, *S. aureus* strain 8325-4 was grown exponentially at 37 °C in LB. SteLL (2 × MIC or 8 × MIC) or ciprofloxacin (1 × MIC) were added and samples were taken for DAPI (4′,6-diamidino-2-phenylindole) staining after 3 h. The images were recorded by fluorescence microscopic system (Axio Imager Z2, Carl Zeiss, Germany).

### SOS response assay

The induction of SOS response was measured using a derivative *S. aureus* 8325-4 strain carrying a *recA::lacZ* fusion. Bacteria were grown exponentially in LB medium until an OD_600_ between 0.1 and 0.2. Cells were treated with SteLL or ciprofloxacin (both at 0.5 × MIC) for 3 h^70^. Cells were permeabilizated by toluene and β-galactosidase activity was measured using ONPG (ortho-Nitrophenyl-β-galactoside; Sigma-Aldrich).

### Staphyloxanthin inhibition assay

Overnight cultures of *S. aureus* ATCC 29312 (a strong staphyloxanthin producer) were diluted (1:100) in LB medium and samples (1 mL) of this suspension were incubated with sub-inhibitory concentrations of SteLL (0.0625 × MIC, 0.125 × MIC, 0.25 × MIC, 0.5 × MIC). After overnight incubation at 37 °C, the tubes were centrifuged (9000 × *g* for 10 min), suspended with 1 mL of phosphate-buffered saline (PBS) and re-centrifuged. Bacteria cells were then photographed. Next, an assay to quantify carotenoid pigments (including staphyloxanthin) was performed. For this, each pellet was resuspended in methanol (0.2 mL) and incubated for 3 min at 55 °C. The methanol phase (supernatant) and cell debris were separated by centrifugation (9000 × *g* for 10 min) and the pellets were submitted to entire pigment extraction procedure three more times. Finally, the absorbance of methanol extract was determined at 465 nm^34^.

### Assays with mice peritoneal macrophages

#### Isolation of mice peritoneal macrophages

Peritoneal macrophages were obtained from inbred strains of C57BL/6 mice of both sexes at 8–10 weeks of age. Exudate cells were harvested by peritoneal lavage using 10 mL of ice-cold sterile phosphate-buffered saline (PBS) (pH 7.2). After centrifugation at 120 × *g* for 5 min, the cell pellets were suspended in RPMI-1640 medium supplemented with bovine calf serum (10%; v/v), penicillin and streptomycin (100 U/mL) (all from Sigma-Aldrich). For all assays, macrophages (1 × 10^6^ cells/mL) were cultured in 24- or 96-well plates, and non-adherent cells were removed. All animal experiments were performed according to the ethical standards of the CEUMA University and were approved by the ethics committee for animal experimentation of this institution (CEUA-CEUMA) (Protocol of Approval N° 107/14), which follows the principles of care with laboratory animals.

#### Determination of nitric oxide (NO) production and cell viability

For both assays, macrophages (1 × 10^6^ cells/mL) were seeded in 96-well plates for 24 h at 37 °C and 5% CO_2_. The cells were then treated with SteLL (2, 4, 8 and 16 µg/mL) for another 24 h. Next, the supernatant was used for determination of NO production, and the adherent cells were assessed by the MTT assay (below). Untreated cells were used as negative control. LPS (*Escherichia coli*; 2000 ng/mL; Sigma-Aldrich) + INF-γ (100 ng/mL; BD Pharmingen) were used as inductors of macrophages activation (M1 macrophages), while IL-4 (400 ng/mL; BD Pharmingen) + IL-13 (400 ng/mL; BD Pharmingen) were used for induction of alternative activation (M2 macrophages). The assays were performed following the protocols described below in quadruplicate in two independent experiments. The results are expressed as the mean ± standard deviation (S.D.).

- NO production: The measurement of NO production by peritoneal macrophages was determined using the Griess assay. Briefly, a 50 μL sample from the supernatant of each well was mixed with 50 μL of Griess reagent in a 96-wells plate. After incubation for 15 min at room temperature, the optical density was determined at 540 nm with a microplate reader (Benchmark Plus, Bio-Rad, CA, US). The nitrite concentration (μmol/10^6^ cells) was quantified by extrapolation from a sodium nitrite standard curve for each experiment.
- MTT assay: Cell viability was evaluated using the MTT assay, which measures the metabolic conversion of 3-(4,5-dimethylthiazol-2-yl)-2,5-diphenyltetrazolium bromide (MTT) salt to colored formazan dye. At the end of the incubation period, the medium was removed, and fresh RPMI medium containing 5 mg/mL MTT solution was added, and the sample was incubated for 3 h. Subsequently, the medium was removed, and the intracellular formazan product was dissolved in DMSO. The optical density (OD) was measured at 595 nm. Cell viability was expressed as the % of viable cells compared to the control.

#### Mitochondrial superoxide production

Mitochondrial superoxide anion production by the macrophages was evaluated using a MitoSOX™ Red Mitochondrial Superoxide Indicator (Molecular Probes). Peritoneal macrophages were treated with SteLL (8 and 16 µg/mL) for 30 min. After trypsinization and washing with PBS, MitoSOX™ Reagent (1 mL, 5 μM) was added to the culture samples and incubated for 10 min at 37 °C, protected from light. The cells were washed three times with warm PBS and analyzed using a BD Accuri C6 Flow Cytometer (BD Biosciences, San Jose, CA, USA). The events (at least 10,000) were analyzed with FlowJo 7.6.1 software (TreeStar-Ashland, OR, USA).

#### Macrophage infection and treatment with SteLL

Overnight cultures of *S. aureus* 8325-4 were centrifuged at 10,000 × *g* for 10 min, washed twice with PBS, and resuspended in PBS. In parallel, peritoneal macrophages (1 × 10^6^ cells/mL) were seeded in a 96-wells plate for 24 h and infected with *S. aureus* 8325-4 at a ratio of 10:1 (bacteria/cells) in the presence or absence of lectin (2, 4, 8 and 16 µg/mL). After 24 h, the supernatant was assayed for nitric oxide as described above.

#### Bacterial killing assay

The effects of SteLL on the bactericidal effect of peritoneal macrophages were evaluated towards intracellular and extracellular bacteria. To quantify extracellular bacteria, aliquots of 4 µL of 10-fold-diluted suspensions from cell supernatants were added to agar plates. For the measurement of intracellular bacteria, the supernatants were removed and each well was washed 5× with ice-cold PBS containing trypan blue in order to remove extracellular bacteria. Following, the cells lysed after washing in 0.1 mL sterile water^71^. The cell lysates were 10-fold-diluted and added in agar plates. All plates were incubated at 37 °C for 24 h. After this period, the colonies were counted for the calculation of CFU/mL.

#### Measurement of cytokine release by macrophages

The levels of cytokines (TNFα, IL-6, IL-17A, IFN-γ, IL-4, IL-12 and IL-10) in the supernatant of macrophages were determined by using Mouse cytometric bead array (CBA) cytokine kits (BD Biosciences, Brazil) according with the manufacturer’s instructions. Analysis was performed on a BD Accuri 6 flow cytometer. Results were calculated in CBA FCAP Array software (BD Biosciences, Brazil) and expressed as pg/mL.

### *In vivo* infection model with *Galleria mellonella*

#### Survival assay

*G. mellonella* larvae (~200 mg) were randomly distributed in three experimental groups (*n* = 10). Two groups were infected by injection of 10 μL of a fresh *S. aureus* 8325-4 suspension (1 × 10^5^ CFU/mL) into the last left proleg, followed by incubation at 37 °C. After 2 h, one group of animals received 10 μL of 4 μg/mL SteLL (2 × MIC) dissolved in PBS (resulting in a dose of 0.2 mg/kg). The second group of animals was treated with PBS. The larvae were incubated at 37 °C, and the larval viability was determined daily for 4 days.

#### Quantification of *S. aureus* in *G. mellonella* hemolymph

*G. mellonella* larvae were infected with *S. aureus* and treated as described above. Each day, a total of 5 larvae were cut in the cephalocaudal direction with a scalpel blade and squeezed to remove the hemolymph. Each sample was 10-fold-diluted in PBS and 4 µL was plated on LB agar. After 24 h-incubation at 37 °C, the colonies were enumerated, and the results were expressed as CFU/mL.

### Statistics analysis

All experiments were performed in quadruplicates and in at least two independent assays. Plotting of data was performed using GraphPad Prism 5. Experiments with p < 0.05 were considered significant and are stated in the results section. The survival plots for *in vivo* infection were performed using Kaplan-Meier analysis on pooled data for repetitive experiments. Statistical analysis was carried out with log-rank (Mantel-Cox) test for comparison of survival curves. Experiments with p < 0.05 were considered significant and are stated in the results section.

## Conclusion

Altogether, the findings of this study suggest that the SteLL impairs cell division of *S. aureus* without provoking DNA damage. The lectin also alters bacteria metabolism resulting in a reduced staphyloxanthin production. These effects may be responsible for the anti-infective activity of SteLL in *G. mellonella*. Furthermore, we demonstrate that SteLL increase the production of cytokines by uninfected macrophages and modulate the release of IL-17A and IFN-γ in *S. aureus*-infected macrophages. Taken together, these findings from both *in vitro* and *in vivo* studies suggest SteLL as a promising lead for the development of new anti-infective agents against *S. aureus*.

## Supplementary information

Supplementary Info File #1

## Data Availability

The data used to build all graphs to support the findings of this study are available from the corresponding author upon request.
