# Supplementary material for: Schinus terebinthifolia leaf lectin (SteLL) has anti-infective action and modulates the response of Staphylococcus aureus-infected macrophages
Source: Sci Rep. 2019 Dec 3;9:18159. doi: 10.1038/s41598-019-54616-x (PMC6890730; doi:10.1038/s41598-019-54616-x)
Supplement: Supplementary file 1 — Supplementary Info File #1 [file 41598_2019_54616_MOESM1_ESM.pdf]

**_Schinus terebinthifolia_ leaf lectin (SteLL) has anti-infective action and modulates the response of _Staphylococcus aureus_-infected macrophages**

Isana Maria de Souza Feitosa Lima[1], Adrielle Zagmignan[1], Deivid Martins Santos[1], Hermerson Sousa Maia[1], Lucas dos Santos Silva[1], Brenda da Silva Cutrim[1], Silvamara Leite Vieira[1], Clovis Macêdo Bezerra Filho[2], Eduardo Martins de Sousa[1], Thiago Henrique Napoleão[2], Karen Angeliki Krogfelt[3,4], Anders Løbner-Olesen[5], Patrícia Maria Guedes Paiva[2], Luís Cláudio Nascimento da Silva[1*]

[1]Programas de Pós-Graduação, Universidade Ceuma, São Luís, Maranhão, Brazil;

[2]Departamento de Bioquímica, Universidade Federal de Pernambuco, Recife, Pernambuco, Brazil;

[3]Department of Viral and Microbial Diagnostics, Statens Serum Institut, Copenhagen, Denmark;

[4]Department of Science and Environment, Roskilde University, 4000 Roskilde, Denmark;

[5]Department of Biology, Section for Functional Genomics, University of Copenhagen, Copenhagen, Denmark.

[*]Corresponding author:

Luís C. N. da Silva

Programa de Pós-Graduação em Biologia Microbiana, Universidade Ceuma

Rua Josué Montello, nº 1, Renascença II, São Luís - MA, CEP 65.075-120

E-mail: luiscn.silva@ceuma.br; luisclaudionsilva@yahoo.com.br

**Supplementary Information**

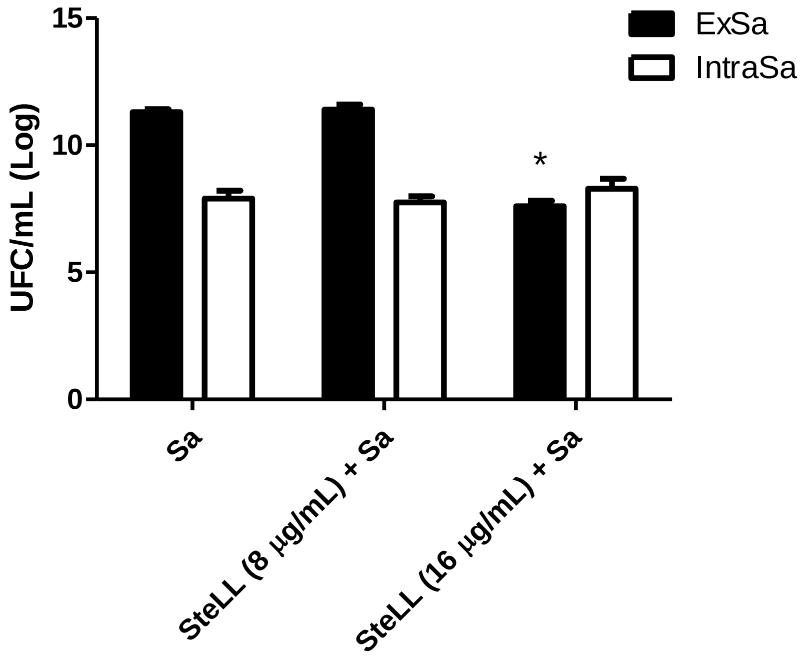


**Supplementary Figure 1:** Effects of SteLL on bactericidal activity of mice peritoneal macrophages towards extracellular and (ExSa) intracellular (IntraSa) *S. aureus* ATCC 6538. M1 macrophages: macrophages treated with LPS + INF-γ. M2 macrophages: macrophages treated with IL-4 + IL-13. Con: untreated cells; Sa: *S. aureus*. (*) Indicates significant differences in relation to *S. aureus*-infected cells without SteLL treatment ($p < 0.05$).
